# Invasive Bacterial Vaccine-Preventable Disease Surveillance: Successes and Lessons Learned in Bangladesh for a Sustainable Path Forward

**DOI:** 10.1093/infdis/jiab129

**Published:** 2021-09-01

**Authors:** Senjuti Saha, Samir K Saha

**Affiliations:** 1 Child Health Research Foundation, Dhaka, Bangladesh; 2 Dhaka Shishu Hospital, Dhaka, Bangladesh; 3 Bangladesh Institute of Child Health, Dhaka, Bangladesh

**Keywords:** surveillance, LMICs, epidemic control, disease burden, infection control, Bangladesh

## Abstract

We have made considerable progress in setting and scaling up surveillance systems to drive evidence-based policy decisions, but the recent epidemics highlight that current systems are not optimally designed. Good surveillance systems should be coordinated, comprehensive, and adaptive. They should generate data in real time for immediate analysis and intervention, whether for endemic diseases or potential epidemics. Such systems are especially needed in low-resource settings where disease burden is the highest, but tracking systems are the weakest here due to competing priorities and constraints on available resources. In this article, using the examples of 3 large, and mostly successful, infectious disease surveillance studies in Bangladesh, we identify 2 core limitations—the pathogen bias and the vaccine bias—in the way current surveillance programs are designed for low-resource settings. We highlight the strengths of the current Global Invasive Bacterial Vaccine Preventable Disease Surveillance Network of the World Health Organization and present case studies from Bangladesh to illustrate how this surveillance platform can be leveraged to overcome its limitations. Finally, we propose a set of criteria for building a comprehensive infectious disease surveillance system with the hope of encouraging current systems to use the limited resources as optimally as possible to generate the maximum amount of knowledge.

Surveillance is the cornerstone of data-driven public health policy decisions for endemic infectious diseases and forecasting epidemics. While in the last 2 decades we have undoubtedly made considerable progress in setting and scaling up surveillance systems to drive evidence-based policy decisions, the successive waves of recent epidemics like Ebola, dengue, Zika, and now coronavirus disease 2019 (COVID-19) highlight how current systems are not yet optimally designed. In theory, good surveillance systems should be coordinated, comprehensive, and adaptive. They should generate real-time data for immediate analysis and intervention, whether for endemic diseases or potential epidemics. Such systems are especially needed in low- and middle-income country (LMIC) settings where disease burden remains high and which are most affected by globalization and climate change. In reality, LMICs tend to have weaker tracking systems due to competing priorities and constraints on available resources.

In this article, using the examples of 3 large, and mostly successful, infectious disease surveillance (IDS) studies in Bangladesh, we identify 2 core limitations in the way current surveillance programs are designed for LMICs. Next, we highlight the strengths of the WHO-coordinated Invasive Bacterial Vaccine-Preventable Disease surveillance system, and how this can be leveraged or repurposed to overcome the 2 core limitations. We present case studies from Bangladesh illustrating how we have successfully done this for optimal use of resources and data generation, and based on our experience, we propose a set of criteria to build a comprehensive IDS system.

## SURVEILLANCE FOR PEDIATRIC PNEUMONIA, MENINGITIS, AND SEPSIS CAUSED BY *STREPTOCOCCUS PNEUMONIAE*, *HAEMOPHILUS INFLUENZAE*, AND *NEISSERIA MENINGITIDIS* IN BANGLADESH

There have been 3 large surveillance studies to detect pneumonia, meningitis, and sepsis caused by *Streptococcus pneumoniae* (pneumococcus) and *Haemophilus influenzae* type b (Hib) in children aged <5 years in Bangladesh. The first was the vaccine alliance Gavi’s Pneumococcal Vaccines Accelerated Development and Introduction Plan (PneumoADIP), which was initiated in 2004 in Bangladesh to assess the burden of pneumococcus and generate evidence for the available pneumococcal conjugate vaccines [1]. Second, in 2008, with Gavi’s support, a separate Hib Initiative surveillance program was rolled out in Bangladesh to generate data on the burden of Hib, against which a vaccine had been available since the 1980s [2]. PneumoADIP-supported surveillance continued in parallel to Hib initiative for a few months in 2008; PneumoADIP ended in 2008 and the Hib Initiative surveillance ended in 2011. Third, in 2009, Gavi initiated a more inclusive surveillance platform called the Global Invasive Bacterial Vaccine-Preventable Disease (IB-VPD) Surveillance Network (GISN), which is coordinated by WHO [3]. This platform gradually evolved and finally included 3 pathogens—pneumococcus, Hib, and *Neisseria meningitidis* (meningococcus), all 3 of which cause a similar spectrum of syndromes. The objectives of GISN were to enroll children <5 years of age admitted in sentinel hospitals to (1) collect data to describe the epidemiology and estimate the burden of the invasive diseases caused by the 3 pathogens; (2) establish a surveillance platform to measure impact after introduction of Hib or pneumococcal vaccine; and (3) detect and characterize the circulating bacterial types [4]. Inclusion criteria are provided in Table 1. Specific information for *H. influenzae*, *S. pneumoniae*, and *N. meningitidis* can be found elsewhere [5–7]. Data are stored only for cases positive for these pathogens of interest.

**Table 1.** Case Definitions for Enrollment in Invasive Bacterial Vaccine-Preventable Disease Surveillance for Meningitis, Pneumonia, and Sepsis

Suspected Case Definition	Inclusion Criteria for Admitted Children
Meningitis	(1) Sudden onset of fever (temperature of >38.5°C rectal or >38°C axillary) and 1 of the following signs: neck stiffness, altered consciousness with no other alternative diagnosis, or other meningeal sign in a child aged 0–59 months, or (2) a clinical diagnosis of meningitis in a hospitalized child aged 0–59 months. OR Patient with cerebrospinal fluid examination showing turbid appearance or leukocytosis (>100 cells/µL) or both leukocytosis (10–100 cells/µL) and either an elevated protein (>100 mg/dL) or decreased glucose (<40 mg/dL). If protein and glucose results are not available, diagnosis is based on turbid appearance or leukocytosis (>100 cells/µL).
Pneumonia	Cough or difficulty breathing and displaying fast breathing when calm at a rate of ≥60 breaths/min in an infant aged <2 months, ≥50 breaths/min in an infant aged 2 to <12 months, or ≥40 breaths/min in a child aged 12–59 months. OR A child 0–59 months old displaying 1 or more of the following: inability to drink or breastfeeding, vomiting everything, convulsions, prostration/lethargy, chest indrawing, stridor when calm.
Sepsis	Presence of at least 2 of the following danger signs and without meningitis or pneumonia clinical syndrome in a child aged 0–59 months: inability to drink or breastfeed, vomiting everything, convulsions (except in malaria-endemic areas), prostration/lethargy (abnormally sleepy or difficult to wake), severe malnutrition, hypothermia (≤36.0°C).

Specific information for *Haemophilus influenzae*, *Streptococcus pneumoniae*, and *Neisseria meningitidis* can be found elsewhere [5–7].

These 3 surveillance systems generated the first comprehensive estimates of pneumococcus and Hib burden in Bangladesh, and facilitated the introduction of the Hib vaccine in 2009 and the 10-valent pneumococcal conjugate vaccine in 2015. Hib vaccine alone is estimated to prevent deaths of 3100 infants every year [8], illustrating the role of these surveillance studies in saving lives of Bangladeshi children. However, they have also been very expensive surveillance systems, and embody 2 core limitations in their designs:

1. The pathogen bias: Surveillance studies in LMICs are conventionally designed to conduct vertical research on specific known pathogen(s) of global interest, often based on historical data from high-income countries. This is despite the fact that multiple different pathogens can lead to clinically indistinguishable syndromes. In a study hospital, a child may present with meningitis or sepsis caused by any of a multitude of pathogens, and a typical microbiological culture of the cerebrospinal fluid (CSF) or blood will yield growth of almost any bacteria in the sample, not only the pathogen of interest. In addition, vertical systems cannot capture emerging pathogens or pathogens that do not exist yet, severe acute respiratory syndrome coronavirus 2 (SARS-CoV-2), being an example. Such top-down approaches not only lead to underutilization of resources in already resource-constrained settings, but more importantly, are missed opportunities to generate valuable data on additional etiologies and/or syndromes. A quintessential example of this loss is perhaps described by the delay of 21 years in introduction of the Hib vaccine in Bangladesh since it was licensed for use in the United States. This delay primarily stemmed from the assumption that Hib is not prevalent in the region, despite some small, local studies depicting its burden [9, 10]; Hib surveillance was not included in the PneumoADIP. Once data from Hib Initiative–supported surveillance, which was set up after some preliminary surveillance studies, became available, the Hib vaccine was introduced quickly.

Another example is the increasing burden of neonatal sepsis and meningitis caused by multidrug-resistant gram-negative bacteria such as *Klebsiella pneumoniae*, *Escherichia coli*, *Acinetobacter baumannii*, and *Pseudomonas aeruginosa*. Although these pathogens are captured during default microbiological culture of blood or CSF in studies designed to detect Hib and pneumococcus, no data are collected, stored, and analyzed by the current surveillance platforms. Moreover, the focus on specific pathogens has meant that data on syndromes are not collected. This is a missed opportunity for forecasting/detecting outbreaks, a key component of surveillance. Tracking a disease (syndromes, case definitions, or diagnosis) in real time, instead of just pathogens, offers the opportunity to monitor trends and patterns that may be driven by causes other than the pathogen(s) of interest. Analysis of such data, for example, can detect even slight increases in meningitis or pneumonia case numbers in a study hospital or country, heralding an outbreak.

2. The vaccine bias: The primary focus of vaccine-preventable surveillance systems often appears to be pathogens for which vaccines are available or are in the pipeline. This had often been driven by interest of donors on specific vaccines. However, data generation for endemic pathogens in LMICs for which no vaccines are currently being considered is also of utmost importance.

The example that perhaps best highlights this issue is typhoid. *Salmonella enterica* Typhi, the cause of typhoid, is endemic in South Asia and the predominant cause of bloodstream infection in children over the age of 2 years. While there was abundant sporadic data documenting its burden in South Asia since the 1990s, no large surveillance study captured information of this important pathogen, leading to a deficiency in understanding its true burden [11]. However, with recent increase in donor interest, and a raging outbreak of extensively drug-resistant *Salmonella* Typhi in Pakistan [12], countries are struggling to generate baseline data to convince national and international policy makers to introduce the vaccine and to be prepared to conduct impact studies. Another example to note would be the vaccines against respiratory syncytial virus (RSV), which are currently in clinical trials in high-income countries; RSV is a dominant cause of childhood pneumonia, but there exists a large gap in systematic data from LMICs despite pneumonia surveillance systems in place.

Finally, there are several vaccines and other interventions like monoclonal antibodies being developed to tackle the increasing burden of gram-negative pathogens and RSV. However, the introduction of these interventions in LMICs might once again be delayed by the vicious cycle of lack of data, which can be collected by leveraging the ongoing platforms.

## CASE FOR IB-VPD SURVEILLANCE

WHO coordinates the IB-VPD surveillance systems across all WHO regions and had data reported from 46 member states with 123 laboratories in 2019 [3]. As in Bangladesh, this surveillance network has helped fill the gaps in data in many LMICs [13]. Its objectives are to document the presence of pathogen-specific diseases, identify circulating serotypes, and measure serotype distribution, with a primary focus on pneumococcus, Hib, and meningococcus, as described above. In the postvaccine era, the network contributes to assessing vaccine impact on the pathogens of interest.

Being one of the largest global surveillance networks, strengths of the IB-VPD surveillance platform are manifold. First, it has established standardized laboratory procedures and guidelines for data collection. Second, the IB-VPD surveillance system does not only collect data on the burden of the pathogen, but also captures details of the circulating pathogens that may influence vaccine choice or design. For instance, the serotype distribution of pneumococcus or meningococcus guides which of the available vaccines is most appropriate for a country, or if new vaccine formulations are required. Third, it has set up a global external quality assessment program to document national, regional, and global laboratory capacities in the IB-VPD pathogen diagnosis. This ensures that data attained from different countries and regions are of acceptable quality and are comparable. Fourth, it ensures delivery of laboratory supplies, maintaining proper cold chains, to the doorsteps of the sentinel laboratories. Considering the difficulties in attaining supplies by many laboratories in LMICs, this is perhaps one of the most crucial strengths of IB-VPD [14]. Much of this has been possible through WHO’s close relationship with country governments and establishment of local and regional offices.

## CASE STUDIES: LEVERAGING THE IB-VPD PLATFORM FOR ADDITIONAL SURVEILLANCE STUDIES

While the central reach of the IB-VPD surveillance platform may be limited in terms of pathogens, the strengths of IB-VPD surveillance allow it to be leveraged to expand its capacity according to a country’s needs. Here we present a few examples of the work we have conducted leveraging the IB-VPD surveillance system.

### Integrating Enteric Fever Surveillance

Between 2012 and 2016, we evaluated the feasibility and sustainability of integrating enteric fever surveillance into the ongoing IB-VPD platform. By adding the simple inclusion criteria of fever 38.9°C (≥102°F) for ≥3 days to the existing criteria for meningitis, pneumonia, and sepsis as detailed in Table 1, we were able capture 94% of all enteric fever cases admitted in an IB-VPD sentinel hospital [15]. This showed that where enteric fever poses a substantial burden, enteric fever surveillance can be successfully and sustainably integrated into the standard IB-VPD surveillance platform at a modest cost.

### Integrating Rotavirus Surveillance

WHO coordinates a separate platform for measuring the burden of rotavirus diarrhea [16], with the objectives of generating pre- and postvaccine data on disease burden, identical to that of the IB-VPD platform. However, only a small proportion of the IB-VPD sites also conduct rotavirus surveillance. In 2012, we conducted a feasibility study to integrate rotavirus diarrhea surveillance into the IB-VPD platform and showed that an integrated platform can achieve almost all the required benchmark indicators related to rotavirus surveillance [17]. Integration of these 2 surveillance platforms, rotavirus and IB-VPD, could ease overall management and reduce costs through co-sharing study personnel and operating costs.

### Integrating RSV and Influenza Surveillance

In 2018, we initiated a study to leverage the ongoing IB-VPD platform to collect nasopharyngeal swab samples from children who meet the WHO case definition of RSV infection [18]. As we were already enrolling children meeting the case definition of pneumonia (Table 1), this could be easily integrated (unpublished). We conduct quantitative polymerase chain reaction assays for detection of RSV and influenza using the same molecular platform that has been set up for the IB-VPD platform for molecular typing of pneumococcus, meningococcus, and *Haemophilus*. It must be noted that the WHO runs a network to monitor the burden of influenza that is independent of the IB-VPD surveillance system [19].

### Integrating Antimicrobial Resistance Surveillance

We have been carefully storing the antimicrobial susceptibility test (AST) results of all pathogens detected through the surveillance platforms. As ASTs are part of regular diagnostics in the sentinel hospital, the only additional cost was of storing the data. Using these data, we have reported trends of antimicrobial resistance in pneumococcus [20, 21] and Hib [10, 22]. In addition, we also collected AST data from the enteric fever study add-on, which led to the first-ever report of azithromycin resistance in typhoidal *Salmonella* and its underlying molecular mechanism [23].

### Integrating Pathogen Genomic Surveillance

Pathogen genomics can provide molecular data that are impossible to gain with only phenotypic tests, and hence is becoming a key component of epidemiological surveillance. However, LMICs lag far behind in this field due to high capital costs and lack of resources and training. We have been maintaining a biobank of all isolates collected through IB-VPD surveillance and its add-on surveillance platforms mentioned above. The historical and living biobank have attracted donors to fund studies to conduct genome sequencing of these stored pathogens. This will help close the knowledge gap about the molecular characteristics of pathogens circulating in Bangladesh and the surrounding regions [24]. Leveraging the genomic sequencing capacity, we were the first to sequence SARS-CoV-2 in Bangladesh [25], and are continuing the SARS-CoV-2 genomic surveillance for the country.

In addition, we have also systematically preserved CSF samples from children who meet the case definition of meningitis, but no microbiological diagnosis could be attained using standard laboratory procedures. We have used these CSF samples, which are linked to clinical data collected through the IB-VPD platform, to conduct unbiased RNA metagenomic studies. Our first metagenomic study revealed several unknown causes of meningitis and uncovered a previously unrecognized chikungunya meningitis in Bangladesh in 2017 [26]. This was the first study establishing the role of chikungunya virus in causing neurological infections in children.

### Integrating Emerging Disease and Outbreak Surveillance

The IB-VPD surveillance platform, like most other infection disease surveillance systems, does not collect data on the total burden of a syndrome, but only of invasive infections caused by specific pathogens. Without disease denominators, it can be difficult to compare temporal trends between and across countries. It also inhibits real-time disease tracking and immediate response to anomalies in trends and patterns. We collect denominators—all patients meeting WHO IB-VPD case definitions, irrespective of whether a pathogen is detected or not. This helps us to monitor and predict trends and seasonality of sample collection and enrollment numbers. This system led to the recognition of a surge of meningitis case numbers in 2017, which later was found to be caused by chikungunya virus outbreak [26].

## TOWARDS BUILDING THE NEXT GENERATION OF INFECTIOUS DISEASE SURVEILLANCE

The most essential steps of setting up a surveillance platform are arguably building relationships with country governments, setting up the infrastructure for systematic collection of patient data in sentinel hospitals, and linking the data to findings from quality-controlled microbiology laboratories. These are also the most difficult, time-consuming, and expensive steps of a multicountry IDS system. WHO-coordinated IB-VPD surveillance network has indeed been able to achieve that in many countries, but maintaining funding for this and other surveillance networks at a global, regional, and country level is quite challenging. In order to sustainably keep up with the current changing world of infectious diseases, it is perhaps time to consider a more comprehensive IDS system, which is in line with WHO’s global comprehensive VPD surveillance [27]. Criteria for such a system are as follows:

- The next generation of IDS systems will include a broader range of infectious disease agents, and not just bacteria. This will require the multiple current infectious disease sentinel surveillance platforms (rotavirus, IB-VPD, influenza, etc) of WHO to connect, share resources, and share costs. If successful, this may reduce internal bureaucracy and make delivery of supplies and funding more efficient and timely.

- For diseases of interest, all pathogens detected in the sentinel microbiology laboratories will be recorded, whether immediately vaccine preventable or not. This will generate data on the diversity of causes of infection and can aid in future vaccine introduction/design.
- The denominator of all cases in the sentinel hospital that meet the case definitions of diseases will be noted. This will allow for real-time tracking of disease trends, noting anomalies. It will also highlight how many cases evade current laboratory testing and hence requirement of new diagnostics.
- AST results will be recorded for bacteria to monitor trends and patterns. The already existing external quality assurance system can be leveraged to assure quality of ASTs.
- The molecular setup for bacterial typing will be leveraged for identification of common viral pathogens such as RSV, influenza, and others.
- Case-linked biobanks will be maintained wherever possible to allow for future genomic studies of circulating pathogens and generate country-specific data.
- The surveillance will be adaptive and responsive to local context. Multicountry surveillance systems often strive to keep design of the programs consistent across countries. While some consistency is undoubtedly required for comparison and quality assurance, it will be remembered that disease and pathogen burden vary geographically. For example, meningococcal meningitis is of high priority in sub-Saharan Africa, but the burden is low in South Asia. Nontyphoidal *Salmonella* is a much bigger problem in Africa than in Asia. Data will be analyzed in real time to guide what pathogens should be prioritized in which countries/regions.

The list is by no means exhaustive of all suggestions, and may vary significantly by region, country, priorities, and funding. Building such a system for IDS, which requires both comprehensive laboratory work and collection of clinical and epidemiological data, would require alignment and coordination between various groups within the donor and coordination teams. And if indeed built, a limitation may be loss of some pathogen-specific data due to a broader focus. In addition, because of increased costs, complexities, and coordination, it might require a fine balance between the number of sites and the quality of data.

One method of achieving this may be by reducing the number of sites in the network to include only high-performing sites and/or sites encountering the highest burden of diseases. However, this might lead to underrepresentation of some countries with the least infrastructure and/or capacity and lead to a vicious cycle of perpetual lack of data from certain countries due to no investment in surveillance. Another method of building a comprehensive surveillance network may be through guiding and equipping the regional reference laboratories to conduct the comprehensive, laboratory-intensive work, which can, in turn, support the sentinel sites in collection of comprehensive data linked to the laboratory work. For example, bacterial isolates/clinical specimens can be shipped to the reference laboratories for identification and additional characterization, and sentinel sites can aid in collection of comprehensive clinical and epidemiological data linked to the specimens.

The core message we want to deliver through this article is that if the limited resources that are available are being spent, we must try to use them as optimally as possible to generate the maximum amount of knowledge. The IB-VPD sentinel sites in Bangladesh also started with very limited resources but have been able to leverage the existing resources optimally to create a virtuous cycle of data generation. We hope to encourage other groups in the global network to join us. In theory, such a holistic IDS platform, without being tied to the attention paid to one pathogen, can be part of a health system that can meet needs of pathogen-specific funders for add-on studies, and provide information about health systems, endemic pathogens, and their variation with geography and time. A good infrastructure for such a surveillance can also prepare sites/countries to forecast and respond to outbreaks, adapt to changing needs, and act as sites for clinical trials or impact studies of new diagnostics, therapeutics, or preventatives in times of need.

## Notes

***Supplement sponsorship.*** The supplement is sponsored by the World Health Organization and the U.S. Centers for Disease Control and Prevention.

***Potential conflicts of interest.*** The authors: No reported conflicts of interest.

Both authors have submitted the ICMJE Form for Disclosure of Potential Conflicts of Interest. Conflicts that the editors consider relevant to the content of the manuscript have been disclosed.

## References

[1] Levine OS, Cherian T, Shah R, Batson A. PneumoADIP: an example of translational research to accelerate pneumococcal vaccination in developing countries. J Health Popul Nutr 2004; 22:268–74. 15609779

[2] Centers for Disease Control and Prevention. *Haemophilus influenzae* (Hib) clinical features. 2020. https://www.cdc.gov/hi-disease/clinicians.html. Accessed 15 December 2020.

[3] World Health Organization WHO IB-VPD and rotavirus surveillance bulletin—October 2020. https://mailchi.mp/14bf45019a44/who-ib-vpd-and-rotavirus-surveillance-bulletin-june-4972714. Accessed 15 December 2020.

[4] Centers for Disease Control and Prevention. Global invasive bacterial vaccine-preventable diseases surveillance—2008–2014. https://www.cdc.gov/mmwr/preview/mmwrhtml/mm6349a4.htm. Accessed 2 September 2020. PMC4584539 25503919

[5] World Health Organization.Vaccine-Preventable Diseases Surveillance Standards: Haemophilus influenzae. https://www.who.int/immunization/monitoring_surveillance/burden/vpd/WHO_SurveillanceVaccinePreventable_05_HaemophilusInfluenzae_R2.pdf?ua=1. Accessed 29 December 2020.

[6] World Health Organization. Vaccine-Preventable Diseases Surveillance Standards: Meningococcus. https://www.who.int/immunization/monitoring_surveillance/burden/vpd/WHO_SurveillanceVaccinePreventable_12_Meningococcus_R2.pdf?ua=1. Accessed 29 December 2020.

[7] World Health Organization. Vaccine-Preventable Diseases Surveillance Standards: Pneumococcus. https://www.who.int/immunization/monitoring_surveillance/burden/vpd/WHO_SurveillanceVaccinePreventable_17_Pneumococcus_R2.pdf?ua=1. Accessed 29 December 2020.

[8] Sultana NK, Saha SK, Al-Emran HM, et al. Impact of introduction of the *Haemophilus influenzae* type b conjugate vaccine into childhood immunization on meningitis in Bangladeshi infants. J Pediatr 2013; 163:S73–8. 23773597 10.1016/j.jpeds.2013.03.033 PMC4629472

[9] Saha SK, Rikitomi N, Ruhulamin M, et al. The increasing burden of disease in Bangladeshi children due to *Haemophilus influenzae* type b meningitis. Ann Trop Paediatr 1997; 17:5–8. 9176570 10.1080/02724936.1997.11747855

[10] Saha SK, Baqui AH, Darmstadt GL, et al. Invasive *Haemophilus influenzae* type B diseases in Bangladesh, with increased resistance to antibiotics. J Pediatr 2005; 146:227–33. 15689914 10.1016/j.jpeds.2004.09.007

[11] Saha S, Islam MS, Sajib MS, et al Epidemiology of typhoid and paratyphoid: implications for vaccine policy. https://academic.oup.com/cid/article/68/Supplement_2/S117/5371225. Accessed 15 December 2020. 10.1093/cid/ciy1124 PMC6405278 30845325

[12] Klemm EJ, Shakoor S, Page AJ, et al. Emergence of an extensively drug-resistant *Salmonella enterica* serovar Typhi clone harboring a promiscuous plasmid encoding resistance to fluoroquinolones and third-generation cephalosporins. mBio 2018; 9:e00105– 18. 29463654 10.1128/mBio.00105-18 PMC5821095

[13] Hasan AZ, Saha S, Saha SK, et al; Pneumococcal and rotavirus surveillance case study group. Using pneumococcal and rotavirus surveillance in vaccine decision-making: a series of case studies in Bangladesh, Armenia and the Gambia. Vaccine 2018; 36:4939–43. 30037484 10.1016/j.vaccine.2018.06.001

[14] Saha S, Saha S, Saha SK. Barriers in Bangladesh. eLife 2018; 7:e41926. 30234484 10.7554/eLife.41926 PMC6147737

[15] Saha S, Islam M, Uddin MJ, et al. Integration of enteric fever surveillance into the WHO-coordinated invasive bacterial-vaccine preventable diseases (IB-VPD) platform: a low cost approach to track an increasingly important disease. PLoS Negl Trop Dis 2017; 11:e0005999. 29073137 10.1371/journal.pntd.0005999 PMC5658195

[16] Agócs MM, Serhan F, Yen C, et al. WHO Global Rotavirus Surveillance Network: a strategic review of the first 5 years, 2008–2012. MMWR Morb Mortal Wkly Rep 2014; 63:634–7. 25055187 PMC5779425

[17] Tanmoy AM, Ahmed AN, Arumugam R, et al. Rotavirus surveillance at a WHO-coordinated invasive bacterial disease surveillance site in Bangladesh: a feasibility study to integrate two surveillance systems. PLoS One 2016; 11:e0153582. 27096958 10.1371/journal.pone.0153582 PMC4838211

[18] World Health Organization. RSV surveillance case definitions. http://www.who.int/influenza/rsv/rsv_case_definition/en/. Accessed 15 December 2020.

[19] World Health Organization. Global Influenza Surveillance and Response System (GISRS). http://www.who.int/influenza/gisrs_laboratory/en/. Accessed 29 December 2020.

[20] Saha SK, Naheed A, El Arifeen S, et al; Pneumococcal Study Group. Surveillance for invasive *Streptococcus pneumoniae* disease among hospitalized children in Bangladesh: antimicrobial susceptibility and serotype distribution. Clin Infect Dis 2009; 48((Suppl 2):):S75–81. 19191622 10.1086/596544

[21] Hasanuzzaman M, Malaker R, Islam M, et al. Detection of macrolide resistance genes in culture-negative specimens from Bangladeshi children with invasive pneumococcal diseases. J Glob Antimicrob Resist 2017; 8:131–4. 28132873 10.1016/j.jgar.2016.11.009

[22] Saha SK, Darmstadt GL, Baqui AH, et al. Direct detection of the multidrug resistance genome of *Haemophilus influenzae* in cerebrospinal fluid of children: implications for treatment of meningitis. Pediatr Infect Dis J 2008; 27:49–53. 18162938 10.1097/INF.0b013e31814d4e55

[23] Hooda Y, Sajib MSI, Rahman H, et al. Molecular mechanism of azithromycin resistance among typhoidal *Salmonella* strains in Bangladesh identified through passive pediatric surveillance. PLoS Negl Trop Dis 2019; 13:e0007868. 31730615 10.1371/journal.pntd.0007868 PMC6881056

[24] Tanmoy AM, Westeel E, De Bruyne K, et al. *Salmonella enterica* serovar Typhi in Bangladesh: exploration of genomic diversity and antimicrobial resistance. mBio 2018; 9:e02112–18. 10.1128/mBio.02112-18 PMC6234861 30425150

[25] Saha S, Malaker R, Sajib MSI, et al. Complete genome sequence of a novel coronavirus (SARS-CoV-2) isolate from Bangladesh. Microbiol Resour Announc 2020; 9:e00568– 20. 32527780 10.1128/MRA.00568-20 PMC7291105

[26] Saha S, Ramesh A, Kalantar K, et al. Unbiased metagenomic sequencing for pediatric meningitis in Bangladesh reveals neuroinvasive chikungunya virus outbreak and other unrealized pathogens. mBio 2019; 10:e02877–19. 31848287 10.1128/mBio.02877-19 PMC6918088

[27] World Health Organization. Global strategy for comprehensive vaccine-preventable disease (VPD) surveillance. https://www.who.int/publications/m/item/global-strategy-for-comprehensive-vaccine-preventable-disease-(vpd)-surveillance. Accessed 29 December 2020.

